# Non-Nutritional Use of Human Milk Part 1: A Survey of the Use of Breast Milk as a Therapy for Mucosal Infections of Various Types in Poland

**DOI:** 10.3390/ijerph16101715

**Published:** 2019-05-16

**Authors:** Karolina Karcz, Mateusz Walkowiak, Julia Makuch, Igor Olejnik, Barbara Królak-Olejnik

**Affiliations:** 1 Department and Clinic of Neonatology, Wroclaw Medical University, Borowska 213, 50-556 Wroclaw, Poland; barbara.krolak-olejnik@umed.wroc.pl; 2 Neonatology and Neonate Intensive Care Students Scientific Association, Wroclaw Medical University, Borowska 213, 50-556 Wroclaw, Poland; mwalkow95@gmail.com (M.W.); julka.makuch@gmail.com (J.M.); 3 Department of Paediatric Bone Marrow Transplantation, Oncology and Hematology, Wroclaw Medical University, Borowska 213, 50-556 Wroclaw, Poland; olejnik@olejnik.x.pl

**Keywords:** breast milk, milk therapy, mucous membranes

## Abstract

The use of home remedies for the treatment of moderately severe ailments is a common practice in the Polish population. Currently, the topic of the potential non-nutritional properties of human milk is attracting the attention of breastfeeding mothers. This study was aimed at understanding lactating women’s knowledge, attitudes, and practices of non-nutritional breast milk on mucous membranes. The study was conducted among lactating women, who filled out a questionnaire consisting of questions about their knowledge and experiences with non-nutritional use of human milk. Statistical calculations were conducted with chi-square test and c-Pearson coefficient. A total of 1187 women were acted on, whereby 768 of respondents claimed to have knowledge of the non-nutritional use of human milk on mucous membranes, whilst 404 of them claimed that they had used at least one method. Among the most frequently used methods were the treatment of rhinorrhea, lacrimal canaliculi obstruction, and conjunctivitis. A correlation between length of breastfeeding (*p* < 0.001) and knowledge of non-nutritional human milk usage in prophylaxis and treatment of mucous membrane inflammation was found. Breastfeeding duration (*p* < 0.001) and parity (*p* < 0.005) were correlated with the application of those methods in practice. Due to a high propensity to testing those methods, parents’ education in the field of possible risks and importance of medical consultations is necessary.

## 1. Introduction

The use of home remedies to treat moderately severe health complaints, including mild infections, is presumed to be a common practice in the Polish population. The popularity of the methods used is primarily due to their easy availability, the opportunity to use substances of natural origin, and the belief that they are devoid of side effects. According to data collected by the Polish Center for Public Opinion Research (CBOS), Poles use the internet for purposes related to health and treatment; among respondents, 50% visit websites concerning health topics, 13% use forums and groups, 69% use the internet to look for information on how to manage their ailments, and 14% set their own treatment on the basis of information found on the internet, without consulting medical professionals [1].

In recent years, society presented a special interest in uses of human milk for non-nutritional purposes, including therapy. The first attempts to use breast milk as a home remedy appeared hundreds of years ago, and opinions about its properties and treatment effects were passed down from generation to generation [2,3]. Currently, the topic of potential non-nutritional properties of human milk often attract the attention of breastfeeding mothers. The direct source of data supporting the foregoing theses were mothers who consulted the authors’ Clinic of Neonatology on the safety of using their own breast milk in the treatment of rhinitis or conjunctivitis in their offspring. Widely available sources of knowledge, the press and social media, give numerous possibilities for the use of breast milk, especially in the aspect of alternative methods of treatment and prophylaxis. While in lay literature reports of anecdotal effectiveness of the proposed methods can be found, reliable scientific data developed in accordance with the principles of evidence-based medicine are scarce.

The composition of breast milk is optimally adapted to the needs of the newborn infant, in terms of both nutrients and biologically active constituents [4]. These affect the immune status of infants by not only providing protection, but also facilitating development of the immune system, tolerance of antigens, and stimulation of an adequate inflammatory response to contact with pathogens. The beneficial effects of breastfeeding include prevention of gastrointestinal and respiratory tract infections, and reduced risk of atopic dermatitis and the occurrence of autoimmune diseases, such as diabetes type 1, celiac disease, asthma, rheumatoid arthritis, or multiple sclerosis [5,6]. In addition, a mother’s milk contains leukocytes and numerous antimicrobial compounds, including immunoglobulins, acute phase proteins, mediators of cellular communication, lysozyme, lactoferrin, lactoperoxidase, oligosaccharides, and prebiotics [7]. The presence of multiple bioactive components gives rise to the potential of the use of breast milk for therapeutic application.

According to the authors’ knowledge, there are no statistical data on the use of human milk for non-nutritional purposes in Poland. The only identified paper was written in a Polish review of selected worldwide research articles concerning the use of the breast milk in the treatment of neonatal conjunctivitis, breast inflammation, atopic dermatitis, and diaper dermatitis, in the care of umbilical cord swab, and in anticancer therapy as a future perspective [8]. Therefore, the subject of the study was to explore the knowledge of, attitude to, and practices of non-nutritional breast milk use by women during lactation. The aim of this study was to assess the prevalence of the use of their own milk by mothers in the prevention and treatment of ear, ocular, pharyngeal, and nasal mucosa ailments. In this paper, the authors present the results of the survey and provide a brief summary of the representative studies to highlight findings that are considered important.

## 2. Materials and Methods

A cross-sectional study was conducted in November/December 2018 using an anonymous web questionnaire prepared in Polish. Lactating women were asked to complete the survey—both breastfeeding and expressed milk-feeding, regardless of lactation duration. The questions included in the questionnaire were related to contact with information on non-nutritional properties of human milk, their practical application, evaluation of results, and respondents’ willingness to test potentially therapeutic uses of their own milk. The list of methods concerning the non-nutritional use of human milk was based on information found on web forums, blogs, social networks, and in lay literature. 

It needs to be emphasized that, both prior to and after completing the questionnaire, respondents were informed of the non-educational purpose of the survey—the notice on the origin of listed non-nutritional breast milk usages and their scientifically unverified status was posted together with the link to the survey, as well as in its final part (Figure S1, Supplementary Materials).

The questionnaire was posted on Polish-language forums and groups discussing topics of parenthood and breastfeeding, mainly based on Facebook. The groups and online forums were randomly selected, found with a search engine using keywords in Polish such as “lactation”, “breastfeeding”, “mother”, and “parenthood”. The respondents answered anonymously and did not receive any reward. The responses were coded automatically due to the order of reception. The statistical correlation between the variable categories was assessed using the chi-square test. The c-Pearson coefficient was used to determine the relation between nominal variables. The statistical significance level of findings was set at *p* < 0.05. For calculations, PQStat 1.6.6 (PQStat Software Tomasz Więckowski, Plewiska, Poland) and Microsoft Excel for Office 365 (Microsoft, Redmond, WA, USA) were used.

Participation in the survey was entirely voluntary, and, by filling in the questionnaire, women agreed to participation in the study. Prior to the start of the study, formal permission was obtained from the local ethics committee, the Bioethical Committee at the Medical University in Wroclaw (Nr KB 703/2018, 22 November 2018).

## 3. Results

The questionnaire was completed by 1218 mothers, of whom 31 were excluded due to the lack of current lactation during the period of research. The analysis included responses from 1187 women, whereby 890 (74.98%) of the respondents admitted that they had contact with information about non-nutritional uses of breast milk, of which 768 (86.29%) had heard about its therapeutic effect on mucous membranes (Table 1).

**Figure 1:**
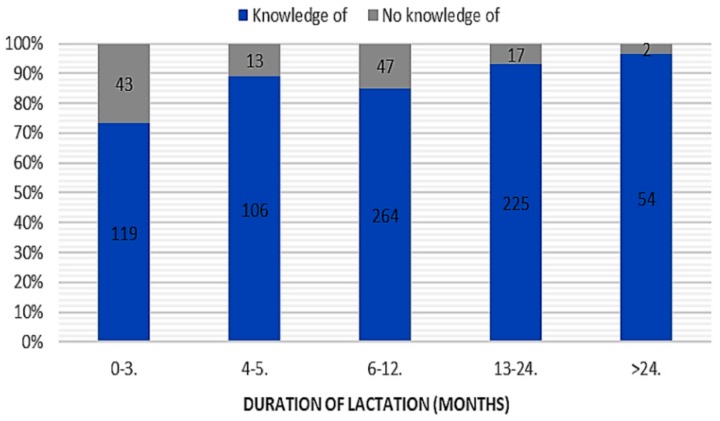
The duration of lactation and “knowledge” of the use of breast milk in prevention and treatment of mucosal ailments (“knowledge of” vs. “no knowledge of”).

**Figure 2:**
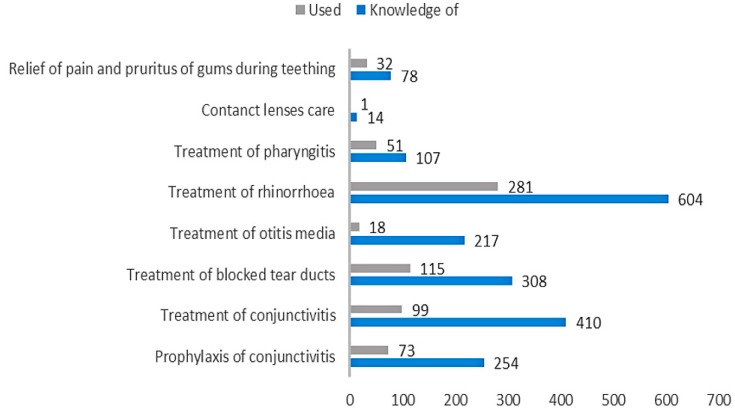
Comparison of the number of mothers who had “knowledge” of using human milk on mucous membranes (“knowledge of”) and the number of mothers who tried it in practice (“used”).

A relationship between duration of lactation (chi-square test = 37.86; *p* < 0.001; c-Pearson adjusted coefficient = 0.286) and “knowledge” of the use of breast milk as a remedy in prophylaxis and treatment of mucosal ailments was found (Figure 1). No significant relationship in terms of place of residence, parity, or gestational age was found. Most often, mothers knew about the use of human milk in treatment of common cold (*n* = 604), conjunctivitis (*n* = 410), and blocked tear ducts (*n* = 308) (Figure 2).

**Figure 3:**
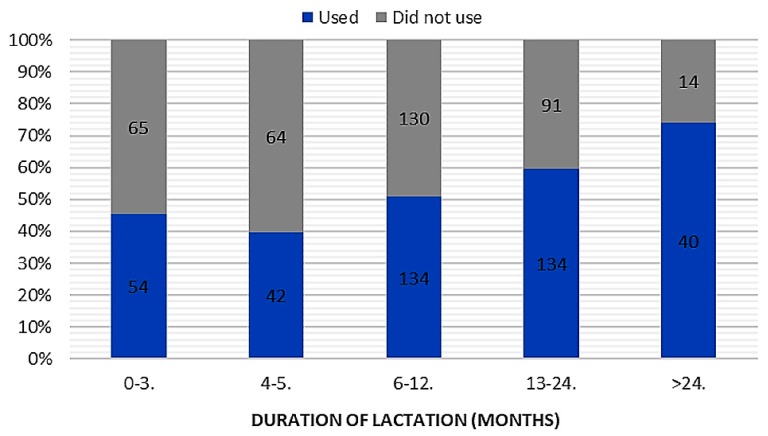
The duration of lactation and attempts to use breast milk in prevention and treatment of mucosal ailments (“used” vs. “did not use”).

**Figure 4:**
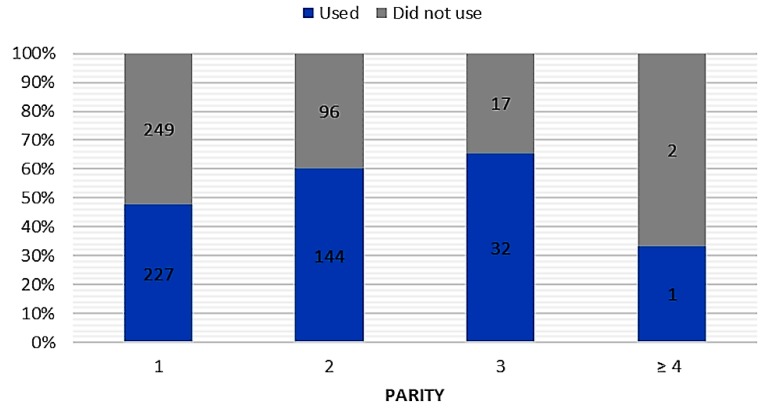
The parity and attempts to use breast milk in prevention and treatment of mucosal ailments (“used” vs. “did not use”).

In total, 404 women implemented knowledge about the use of breast milk on mucous membranes in practice (Table 1). Respondents assessed the results of breast milk use on mucous membranes as positive (339 opinions), negative (34 opinions), or difficult to assess (41 opinions). Among mothers, the most popular applications of their own milk comprised treatment of cold (*n* = 281), blocked tear ducts (*n* = 115), and conjunctivitis (*n* = 99) (Figure 2). In the case of attempts to use breast milk as a home remedy for mucosal complaints, we found a relationship between breastfeeding duration (chi-square test = 24.36; *p* < 0.001; c-Pearson adjusted coefficient = 0.248) and parity (chi-square test = 13.495; *p* < 0.005; c-Pearson adjusted coefficient = 0.186) (Figure 3 and Figure 4).

**Figure 5:**
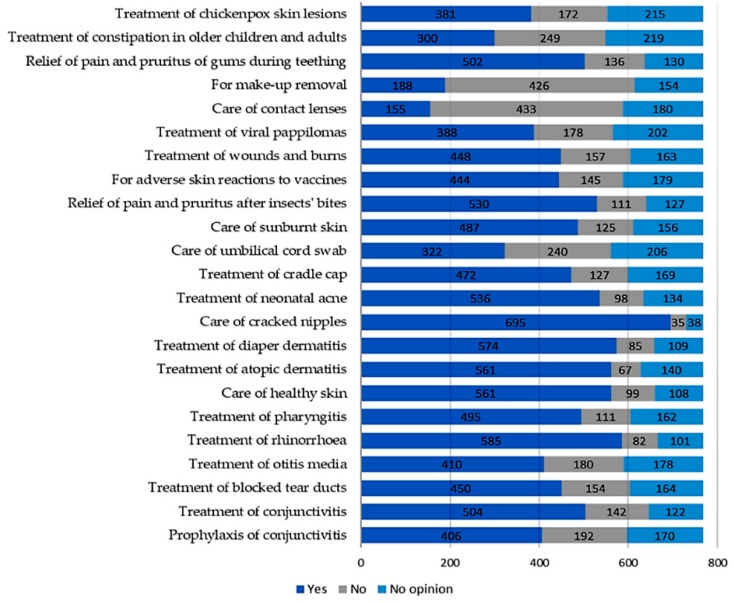
Declared willingness to use selected non-nutritional uses of breast milk in the future. The list of methods was based on information found on online discussing groups, blogs, social networks, and in lay literature. It was remarked that little or no scientific data are currently available regarding the efficacy and dangers of implementation of those usages.

Among the respondents, 14 women discovered information concerning the possibility of cleaning contact lenses with women’s milk. Only one of them decided to put this method into practice, with negative effect—pruritus and redness of the conjunctiva. Furthermore, 171 mothers expressed a willingness to use their own milk for contact lens care in the future (Figure 5).

Among all respondents who applied their milk for non-nutritional purposes (*n* = 751), 63 (8.4%) mothers used milk only on mucous membranes, 347 (46.2%) only on the skin, and 341 (45.4%) in both cases. These three groups were compared with regard to duration of pregnancy, breastfeeding, parity, education, place of residence, and age. A statistically significant relationship was found in terms of (successively increasing correlation) education (chi-square test = 12.77; *p* < 0.05; c-Pearson adjusted coefficient = 0.158), parity (chi-square test = 17.65; *p* < 0.01; c-Pearson adjusted coefficient = 0.186), and breastfeeding duration (chi-square test = 31.24; *p* < 0.001; c-Pearson adjusted coefficient = 0.245).

**Figure 6:**
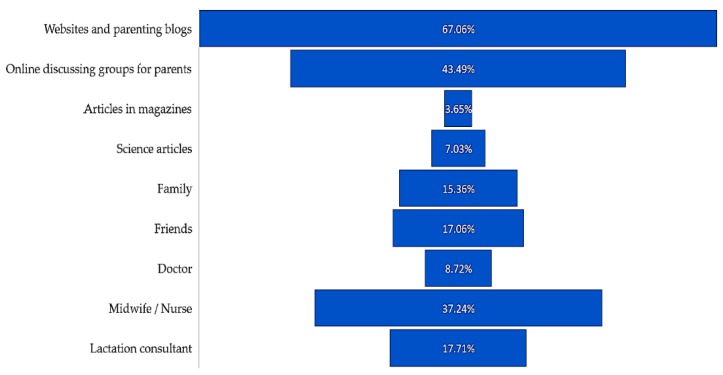
Source of mothers’ knowledge about the possibilities of non-nutritional use of breast milk (multiple choice question).

The basic sources of mothers’ knowledge about the possibilities of non-nutritional use of their own milk were websites (67.06%), online discussing groups for parents (43.49%), and midwives or nurses (37.24%) (Figure 6).

## 4. Discussion

A mother’s milk is an extremely complex fluid, which not only nourishes babies, but protects them from diseases [6,7,9]. Numerous antimicrobial and immunomodulating compounds, including human milk oligosaccharides, compensate for the functional immaturity of the infantile immune system. The bioactive agents which comprise human milk are capable of inhibiting inflammatory processes, as well as increasing the production of specific antibodies, antioxidants, interleukins, growth factors, secretory leukocytes, and defensins. A mother’s milk also contains factors that can mediate differentiation and development of B lymphocytes [10]. Due to the increasing knowledge of breast milk composition, the possibility of its use as a therapeutic agent is being investigated. Tests on animal models (New Zealand rabbits and infant rats) showed that the topical application of oligosaccharides contained in human milk hinders the adhesion of pathogens such as *Streptococcus pneumoniae* and *Haemophilus influenzae* to mucous membranes, thus reducing the risk of infection. It was also found that supply of oligosaccharides within 24 h after infection alleviates the symptoms of pneumonia [11]. Similar results were obtained in vitro—oligosaccharides contained in human milk limited adhesion of pathogens to conjunctival epithelium [12] and intestinal mucosa [13].

It appears that, due to easy accessibility, health-promoting properties, and belief in its harmlessness, women’s milk is used as a remedy in “home medicine”. Its use for non-nutritional purposes attracts great public interest, as confirmed in this study.

As mentioned in Section 1, Polish women are, thus, willing to use natural substances to test therapies with their own milk as an alternative to standard treatments. It should be emphasized that knowledge of this topic comes mainly from uncertain sources (websites and forums for parents). The use of methods recommended by other mothers takes place beyond the control of medical personnel. Our attention was given to greater willingness to try out potentially therapeutic milk properties on children than mothers themselves—for example, the readiness to use breast milk for prophylaxis or treatment of conjunctivitis was declared by 2.5 or three times more women than in the case of contact lens care. As we call into question the safety of the use of breast milk as a remedy for mucosal ailments, we provide below a brief summary of available research papers, with reference to each “human milk usage” included in the survey.

The most popular amongst the mothers surveyed was the use of their own milk in the treatment of rhinitis in infants. Women instilled milk from breast directly into children’s nostrils in order to shorten the duration of infection and to improve nasal patency. In the opinion of 86.12% of mothers, the procedure brought a positive effect. No publications were found on the use of human milk as nose drops in the topical treatment of rhinitis. However, scientific publications reported the protective effect of breastfeeding on upper respiratory tract infections and the beneficial development of nasopharyngeal flora [14,15].

The use of human milk as drops is also recommended on social media for otitis media treatment. This method tested by 18 women was assessed positively; however, at the same time, mothers administered other medications. Previously published reviews of studies indicated an increased frequency of otitis media in children due to a short breastfeeding period and the introduction of milk formula before six months of age [16]. Extended breastfeeding seems to reduce the incidence of otitis media in childhood [17].

There is a lack of reliable scientific data verifying the usefulness of breast milk in the local treatment of pharyngitis. Potential benefits might result from the described anti-inflammatory properties of human milk and its protective effect against upper respiratory tract infections [14,18].

Putting drops of breast milk into purulent eyes is a remedy quite often recommended on parents’ forums. Among the respondents, 254 mothers had heard about the use of milk in prevention of conjunctivitis, while 410 mothers had practical experience in its treatment, of which 29% and 24%, respectively, applied this method. The available scientific papers do not explicitly verify benefits of the abovementioned practices. The American Academy of Pediatrics recommends the use of 1% tetracycline ointment, 0.5% erythromycin ointment, or 0.5–1% silver nitrate solution for prophylaxis of anterior eye segment infections in newborns. The standard management of conjunctivitis consists of antibiotic therapy and antivirals; due to the underlying cause, however, conservative treatment is often sufficient [19].

In previous studies, the activity of colostrum against *Staphylococcus aureus* was demonstrated [20,21,22]. The impact of breast milk and antibiotics on bacteria growth was also compared in vitro; antibiotics showed significantly greater efficacy for most strains, except for gonococci [23]. In the aspect of nasolacrimal duct obstruction, one retrospective study was published; the time it took for newborns’ chronic lacrimation resolution was shorter after instilling eyes with milk [24]. Although the results of the studies seem promising, they should be considered carefully. When it comes to conjunctivitis and eye injuries, delay in ophthalmologic consultations and initiation of proper therapy can lead to serious complications—spread of infection to surrounding tissues (endophthalmitis, panophthalmitis), loss of sight, and even indication for eye enucleation [25].

As far as “milk therapy” is concerned, it is important to note that human milk was generally considered sterile; however, in recent years, it proved to be a permanent source of commensal, mutualist, and potentially probiotic bacteria colonizing the infant’s intestines. The mammary gland contains its own microflora not only during lactation, but in the final stage of pregnancy. The composition of microbiota may vary depending on the duration of pregnancy, type of delivery, maternal weight, duration of lactation, maternal health, and inhabited latitude. Despite these factors, the core microbiota of human milk contains genera *Staphylococcus*, *Streptococcus*, *Serratia*, *Pseudomonas*, *Corynebacterium*, *Propionibacterium*, *Lactobacillus*, and to a lesser extent *Enterococcus* and *Bifidobacterium* [26]. Transitional flora is not without significance. Like any other human body secretion, milk can be a carrier of pathogens—viruses, e.g., HIV, HTLV-1, CMV, HBV, HCV, HSV, VZV; protozoa, e.g., *Toxoplasma*; fungi, e.g., *Candida spp.*; and bacteria, including *Listeria monocytogenes*, *Coxiella burnetii*, *Staphylococcus aureus*, and *Streptococcus B*; cases of milk-transferred infections in infants were also reported [27]. Therefore, safety of human milk application to mucous membranes should be carefully considered, especially in newborns and infants.

The newborn’s microflora is highly dynamic and undergoes rapid changes during the first years of life, striving for a stable structure of distinct microbial communities with unique composition and functions in specific areas of the body. Early interactions between the developing microbiome, pathogenic bacteria, and their human host are responsible for maturation of the immune system in the postpartum period, which affects health in the future [28]. Although the influence of breastfeeding on intestinal microbiota development and upper respiratory tract bacterial flora is well known, it is not clearly defined whether topical application of human milk to mucous membranes, e.g., ocular or nasal, brings health benefits. It is generally recognized, however, that microbial dysbiosis in anatomical niches may lead ultimately to immune dysregulation [15,16,17]. From this standpoint, further research on the topic of “breast milk therapy” for mucosal infections is needed.

## 5. Strengths and Limitations

This paper is a part of the first study, to the authors’ knowledge, on the subject of breast milk use for non-nutritional purposes in Poland. It provides preliminary data on the prevalence of using human milk as a home remedy in the treatment and prevention of mucous membrane complaints, and also includes patients’ experiences. The survey was completed by women from various regions of Poland, differing in terms of age, parity rate, and education. However, as the study was limited mostly to mothers active on social networks, the study group cannot be considered as representative of the Polish population.

## 6. Conclusions

The following conclusions were drawn in this study:

1. A high interest in the non-nutritional applications of breast milk was demonstrated among breastfeeding Polish mothers. Due to the high levels of readiness to use in practice methods recommended on online forums, there is a need to educate parents about possible risks and to highlight the importance of medical consultation in order to initiate adequate treatment.

2. Anecdotal evidence, based on patients’ personal experience, confirming the effectiveness of human milk in the prevention and treatment of mucous membranes ailments is a sufficient argument for mothers to use the methods found on the internet.

3. There is a need for reliable information verifying health benefits and risks associated with the use of breast milk for therapeutic purposes in mucous membranes. Further scientific research in this area should be carried out.

## Figures and Tables

**Table 1:** Characteristics of the population of women who had contact with information about use of human milk for mucous membranes (“knowledge of”) or used it in practice (“used”).

Demographic Data	Knowledge of, *n* = 768 (86.29%)	Used, *n* = 404 (45.39%)
Age in years (Mean ± SD)		
	30.18 ± 3.89	30.32 ± 4.01
Place of residence (*n*)		
Rural	137 (17.84%)	81 (20.05%)
City < 100,000 residents	181 (23.57%)	101 (25.0%)
City > 100,000 residents	450 (58.59%)	222 (54.95%)
Education (*n*)		
Primary education	1 (0.13%)	1 (0.25%)
Basic vocational education	7 (9.11%)	3 (0.74%)
General secondary education	108 (14.06%)	60 (14.85%)
Tertiary education	652 (84.9%)	340 (84.16%)
Parity (*n*)		
1	476 (61.98%)	227 (56.19%)
2	240 (31.25%)	144 (35.64%)
3	49 (6.38%)	32 (7.92%)
≥4	3 (0.39%)	1 (0.25%)
Number of currently breastfed children (*n*)		
1	745 (97.0%)	391 (96.78%)
2	22 (2.87%)	12 (2.97%)
3	1 (0.13%)	1 (0.25%)
≥4	0	0
Duration of lactation in months (Mean ± SD)		
	11.2 ± 8.68	12.65 ± 9.56
Gestational age in weeks (Mean ± SD)		
	39.18 ± 2.1	39.12 ± 2.13

## Supplementary Materials

The following are available online at https://www.mdpi.com/1660-4601/16/10/1715/s1. Figure S1: Introduction and conclusion of the online form used for the survey. A copy of the pre-filled form is available online: https://docs.google.com/forms/d/e/1FAIpQLSewcxKIW39bsUz46dBC6GNXfhXtmSNPowPDniNDpdKbRGq-jw/viewform.
