# Supplementary material for: Non-Nutritional Use of Human Milk Part 1: A Survey of the Use of Breast Milk as a Therapy for Mucosal Infections of Various Types in Poland
Source: Int J Environ Res Public Health. 2019 May 16;16(10):1715. doi: 10.3390/ijerph16101715 (PMC6572138; doi:10.3390/ijerph16101715)
Supplement: Supplementary file 1 [file ijerph-16-01715-s001.pdf]

ORIGINAL QUESTIONNAIRE – INTRODUCTION AND CONCLUSION

Pozażywieniowe zastosowanie mleka kobiecego.

Szanowni Państwo!

Jesteśmy doktorantami i studentami Uniwersytetu Medycznego we Wrocławiu. Zwracamy się z prośbą o wypełnienie kwestionariusza ankietowego, przygotowanego w ramach badania naukowego dotyczącego pozażywieniowego zastosowania mleka kobiecego przez matki karmiące piersią. Adresatami ankiety są Panie karmiące piersią (dopuszcza się karmienie mlekiem własnym przy pomocy np. butelki) - jest to jedyne kryterium niezbędne do uczestnictwa w badaniu.

Niniejsza ankieta jest w pełni anonimowa. Kwestionariusz składa się z 10 pytań, a jego wypełnienie zajmuje ok. 7 minut. Uzyskane wyniki zostaną wykorzystane do przygotowania opracowania naukowego .

Wypełnienie ankiety jest równoznaczne ze zgodą na udział w badaniu.

Bardzo dziękujemy za poświęcenie Państwa czasu!

Zakończenie ankiety.

Jeszcze raz dziękujemy za udział w badaniu.
Informujemy, że uwzględniona w kwestionariuszu lista pozażywieniowych zastosowań mleka kobiecego opracowano na podstawie artykułów zamieszczonych na stronach internetowych i portalach społecznościowych. Nie zostały one zweryfikowane naukowo, a ankieta nie ma charakteru edukacyjnego.

Czy ma Pani jakieś uwagi do ankiety?

TRANSLATED QUESTIONNAIRE – INTRODUCTION AND CONCLUSION

Non-nutritional use of human milk.

Dear Madam!

We are postgraduate students and medical students at Wroclaw Medical University. We kindly ask you to fill out this questionnaire – it was prepared as a part of the study on the non-nutritional use of the human milk by breastfeeding mothers. The survey is addressed to lactating women – both breastfeeding and or expressed milk feeding (e.g. with bottle) – this is the only criterium essential to take part in the study.

The following questionnaire is entirely anonymous. It consists of 10 questions, completing it should take no more than ca. 7 minutes The results obtained will be used to prepare a scientific paper.

Completing the survey also implies your consent to take part in the study.

Thank you very much for taking your time!

Conclusion of the survey.

Once again thank you for taking your time.

We inform that the list of the non-nutritional uses of the human milk included in the questionnaire was prepared on the basis of articles posted on websites and social media. The methods have not beed scientifically verified yet, and the survey has no educational purpose.

Do you have any concluding remarks concerning the survey?
